# Improving the precision of maternal, newborn, and child health impact through geospatial analysis of the association of contextual and programmatic factors with health trends in Bihar, India

**DOI:** 10.7189/jogh.12.04064

**Published:** 2022-11-23

**Authors:** Safa Abdalla, Emma Pair, Kala Mehta, Victoria Ward, Tanmay Mahapatra, Gary L Darmstadt

**Affiliations:** 1 Department of Pediatrics, Stanford University School of Medicine, Stanford, California, USA; 2 Department of Epidemiology and Biostatistics, University of California San Francisco, San Francisco, California, USA; 3 CARE India, Patna, India

## Abstract

**Background:**

There is a scarcity of research that comprehensively examines programme impact from a context-specific perspective. We aimed to determine the conditions under which the Bihar Technical Support Programme led to more favourable outcomes for maternal and child health in Bihar.

**Methods:**

We obtained block-level data on maternal and child health indicators during the state-wide scale-up of the pilot Ananya programme and data on health facility readiness, along with geographical and sociodemographic variables. We examined the associations of these factors with increases in the levels of indicators using multilevel logistic regression, and the associations with rates of change in the indicators using Bayesian Hierarchical modelling.

**Results:**

Frontline worker (FLW) visits between 2014-2017 were more likely to increase in blocks with better night lighting (odds ratio (OR) = 1.23, 95% confidence interval (CI) = 1.01-1.51). Birth preparedness increased in blocks with increasing FLW visits (OR = 3.43, 95% CI = 1.15-10.21), while dry cord care practice increased in blocks where satisfaction with FLW visits was increasing (OR = 1.52, 95% CI = 1.10-2.11). Age-appropriate frequency of complementary feeding increased in blocks with higher development index (OR = 1.55, 95% CI = 1.16-2.06) and a higher percentage of scheduled caste or tribe (OR = 3.21, 95% CI = 1.13-9.09). An increase in most outcomes was more likely in areas with lower baseline levels.

**Conclusions:**

Contextual factors (eg, night lighting and development) not targeted by the programme and FLW visits were associated with favourable programme outcomes. Intervention design, including intervention selection for a particular geography, should be modified to fit the local context in the short term. Expanding collaborations beyond the health sector to influence modifiable contextual factors in the long term can result in a higher magnitude and more sustainable impact.

**Registration:**

ClinicalTrials.gov: NCT02726230.

The Sustainable Development Goals (SDG) have revolutionised the priorities for improving population health and addressing health inequalities, underscoring the role of social determinants of health and the interconnectedness of health with the wider social and economic context [1]. Health programme evaluations, however, typically average out potentially heterogeneous impacts in smaller geographical units that can be influenced by their varying local social and economic contexts and fail to inform context-responsive and targeted programming.

Understanding the context in which health programmes are implemented can be critical for explaining the heterogeneity of programmatic impacts and identifying factors that could maximise or impede the achievement of desired goals. Many of these factors span social determinants that are not necessarily the target of health interventions nor included within the remit of programme implementers [2]. Identifying important contextual factors can create opportunities to adjust programme targets, the interventions chosen for implementation, and implementation methods, and could inform the modification of contextual factors to remove barriers to programme coverage and effectiveness. Contextual analysis is also helpful for understanding what works where and predicting whether an intervention is likely to succeed in a particular setting. By providing insights into the prerequisites for optimal outcomes, it becomes possible to close the gap between programme theory and assumptions and the reality on the ground [3], shift from a limited focus on scaling-up interventions to scaling-up approaches with innovative context-sensitive modifications, and identify potentially marginalised sub-groups in need of targeted programming to advance equity and health impact.

The dearth of research that comprehensively examines programme impact through a context-specific perspective was recently highlighted for interventions that employ community health workers (CHWs), with the authors noting that “future health policy and systems research should better address the complexity of contextual influences on programmes” [4]. Some studies in Asia and sub-Saharan Africa examined the contextual determinants of change in maternal and child health programme outcomes, such as poverty, education, and infrastructure [5-7]. A few studies did the same at area level, revealing, for instance, that introducing financial incentives in rural north India resulted in a higher increase in institutional deliveries where good access to childbirth services was available [8], and that an insecticide-treated bed net campaign in Democratic Republic of Congo reduced all-cause child mortality the most in areas of high malaria transmission risk [9]. However, there is a lack of studies geospatially examining the associations between contextual and programmatic factors with trends in maternal and child health indicators during implementation.

We extended this approach by studying the impact of the Bihar Technical Support Programme (BTSP). We applied geospatial analysis to area-level trends in the *Ananya* programme outcomes in Bihar, a state in eastern India. The programme is described in detail elsewhere [10-13]. Briefly, it piloted a range of interventions through several initiatives by multiple partners to identify and support governmental scale-up of the most effective reproductive, maternal, newborn, and child health and nutrition (RMNCHN) interventions. The initiatives included interventions to increase the knowledge of frontline workers (FLW) and their interactions with beneficiaries, quality improvement (QI) efforts in public health facilities, the use of media campaigns and m-health tools to increase the adoption of priority health behaviours and to improve FLWs’ communications with beneficiaries, and the integration of health modules into traditional self-help groups (SHGs) [14-18]. The first phase of the programme relied on intensive support for governmental implementation of initiatives in eight “focus” districts [11]. The second phase, from 2014 to present, which is the focus of this study, aimed to increase coverage and service quality state-wide in 38 districts through health system strengthening and performance management by providing planning and policy technical support, removing bottlenecks, and building capacity via increased data utilisation [13]. This scale-up phase relied on the government’s own capacity and actors to sustain the interventions piloted in the previous phase.

We previously examined geographic variations at block level – the smallest administrative unit – in levels and trends of 22 RMNCHN indicators from 2014-2017, finding evidence of variations in trends at the block level in 19 of those indicators from across the continuum of care [19]. A programme equity evaluation had shown differential trends in indicators by degree of social and economic marginalisation related to limited health care access, pointing to possible contextual barriers such as remoteness of health facilities with limited access to affordable transportation [20]. Building on that work, we aimed to use geographical variability in contextual factors and indicator trends to understand the conditions under which programme outcomes were more favourable and to determine whether contextual factors influenced and/or interacted with programme processes. In conjunction with qualitative studies, such understanding can inform the fine-tuning of intervention design and implementation and the expansion of multi-sectoral partnerships to influence contextual factors outside the remit of the health system, informing the optimisation of long-term programme impact. We sought to answer the following questions: 1) what factors were associated with improvements in RMNCHN indicator levels between 2014-2017 in Bihar, and 2) what factors were associated with the rate of change in indicator levels between 2014-2017?

## METHODS

### Conceptual Framework

**Figure 1:**
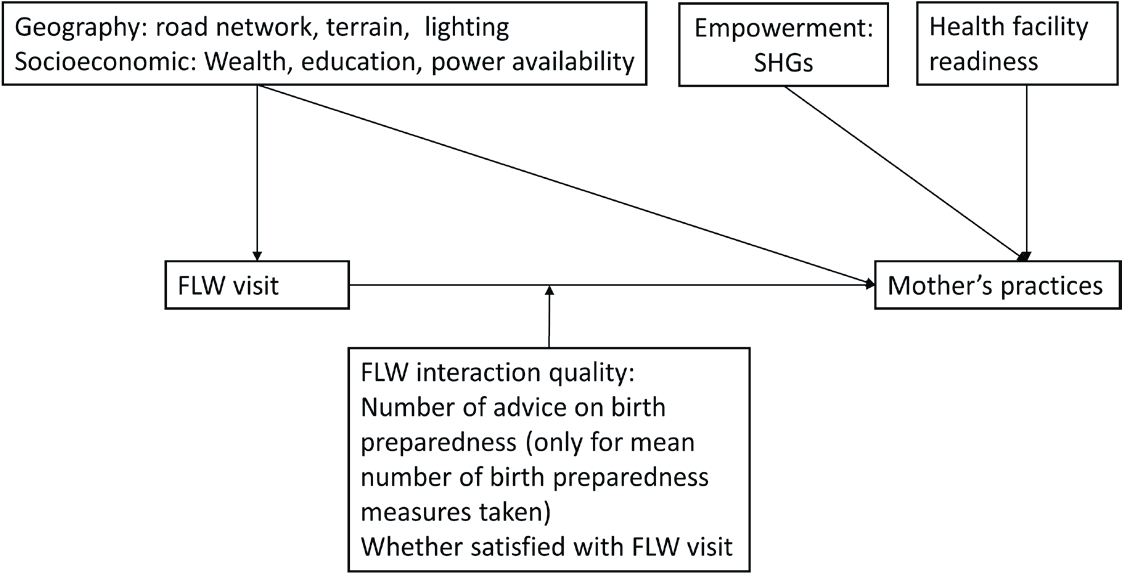
Conceptual framework for the analysis. FLW – frontline worker, SHG – self-help groups

We initially constructed a conceptual framework based on evidence from cross-sectional studies [2,4,21-25]. We limited the final framework (**Figure 1**) to only those components for which we could access block-level data from various sources for the years preceding or overlapping with the study years. Due to the lack of geospatial data on programme inputs such as human resources and finances, we used frontline worker (FLW) visits as an indicator of the programme’s ability to translate inputs into processes. The conceptual model had three components: 1) hypothesised association of contextual factors with changes in FLW visits, 2) hypothesised association of contextual factors with changes in maternal practices, and 3) hypothesised effect modification of the association between FLW visits and change in mothers’ practices by contextual factors and satisfaction of beneficiaries with FLW visits.

### Data

#### The Socioeconomic High-resolution Rural-Urban Geographic Platform for India

The Socioeconomic High-resolution Rural-Urban Geographic Platform (SHRUG) is an open-access repository of data sets on geographic and social factors covering India’s 500 000 villages and 8000 towns using a set of common geographic identifiers that span 25 years [26]. The SHRUG v1.5 was updated in September 2020 and includes several data sources used in the present work (**Table 1**). We averaged the village/town-level SHRUG data to obtain block-level measures.

**Table 1:** Socioeconomic High-resolution Rural-Urban Geographic data sets used for geospatial analysis of association of contextual factors with health in Bihar, India*

Title	Description	Data year used in the study
Population Census data (1991-2011)	Extensive demographics and a detailed listing of public goods for all towns and villages in India for the years 1991, 2001, and 2011.	2011
All-India Forest Cover (Vegetation Continuous Fields) (2000-2019)	Forest cover data derived from tree cover measured at 250-m resolution by Vegetarian Continuous Fields, a MODIS product, from 2000-2019. A machine-learning algorithm derived from broad spectrum satellite images and trained with human-categorised data predicted VCF. It can distinguish between crops, plantations, and primary forest cover.	2014
All-India Night Lights (1994-2013)	National Oceanic and Atmospheric Administration (NOAA) gridded night lights data, available annually from 1992-2013, were matched to village and town polygons and aggregated into totals and means. Estimates were calibrated for consistency among all years. Night lighting is often used as a proxy for economic activity when time series data on this is unavailable.	2013
Pradhan Mantri Gram Sadak Yojana	Administrative data on national rural road construction at the village-level. Includes road completion dates, completion time, costs, overruns, construction materials, and beyond.	2011

*Adapted from “Available Data” by the Data Development Laboratory, 2020, (http://www.devdatalab.org/shrug_download/).

#### Comprehensive Facility Assessments

Comprehensive Facility Assessments (CFAs) were cross-sectional assessments of structural elements, human resources, and essential drugs and equipment to identify gaps in Bihar health facilities' readiness for RMNCHN service delivery, implemented by CARE India. Over 530 facilities from the 38 districts of Bihar were included. The types of facilities included in the study were 1) primary health centres, 2) referral hospitals (community health centres/first referral units/referral hospital (RH)), 3) sub-district hospitals, and 4) district hospitals. The Facility Readiness Score ranges from 0-100 and is the average of 24 indicators covering human resources, maternal health, asphyxia management (newborn care), infection control management, neonatal health, and post-partum haemorrhage management. If a block had more than one listed facility, the facilities’ results were averaged to obtain the block-level score. If facilities in the block had missing data, only the facility with complete information was used. The trend in facility readiness was calculated as the difference between 2015-2017 (2015 was the first available year), which corresponded to the time frame over which we examined changes in RMNCHN indicators.

#### Community-based Household Surveys

The Community-based Household Surveys (CHS) are cross-sectional surveys designed to provide precise estimates for key RMNCHN indicators at the district-level and to track changes in those indicators over time (see indicator definitions in Table S1 in the **Online Supplementary Document**). The methodology was described in detail elsewhere [13]. We previously estimated block-level trends with 95% credible intervals for each indicator using hierarchical Bayesian modelling [19]. For the first research question, we defined an increase in indicator level as a 95% lower credible limit that was above 1. For the second research question, we used the crude estimates in further modelling as described below.

### Variables

**Table 2** describes each of the variables in the conceptual model. We first examined the correlation of all variables in the conceptual model with each other (Figure S1 in the **Online Supplementary Document**). Noting moderate to strong correlations amongst the six contextual variables from the SHRUG database, we subjected them to a principal component analysis, retaining two components (Table S2 in the **Online Supplementary Document**). Percentage of the population who were literate, percentage with power available for domestic use in either summer or winter, and average lighting loaded on the first component, which we labelled “Development”. Average distance to the nearest town with population ≥500 000, average forest cover, and percentage of land with paved roads loaded on the second component, which we labelled “Landscape”. We used the two indices in regression models; if no significant association was found, we further tested their component variables individually.

**Table 2:** Description of block-level independent and dependent variables in the conceptual model for analysis of the association of contextual and programmatic factors with trends in reproductive, maternal, newborn, and child health and nutrition in Bihar, India

Variable	Source	Description
**Independent**		
Remoteness	SHRUG	Distance to the nearest town with population ≥500 000 population for each village, averaged over all villages in each block.
Night lighting	SHRUG	Sum of the night-time luminosity values (0-63) of all pixels in each village in 2013, averaged over all villages in each block
Forest coverage	SHRUG	Sum of total forest pixel values in each village in 2014 averaged over all villages in each block. Each Vegetation Continuous Fields pixel takes a value of 0 to 100, reflecting the percent of that pixel covered by forest. The sum of the percentages over all village pixels can therefore exceed 100.
Literacy	SHRUG	Percentage of the population in each block who knew how to read and write in 2011.
Power availability for domestic use	SHRUG	Total daily hours of power available for domestic use during the summer and winter in each village in 2011, averaged over all villages in each block.
Land with paved roads	SHRUG	Percentage of villages/towns in a block that had any paved roads in 2011.
Belonging to scheduled caste or tribe	SHRUG	Percentage of the population in each block which belonged to a scheduled caste or tribe in 2011.
Trend in facility readiness score	CFA	Difference between Facility Readiness Scores in 2015 and 2017.
Trend in asset ownership	CHS	Annual change in log average number of assets owned per block between 2014-2017.
Trend in self-help group membership	CHS	Annual change in log-odds of membership of a SHG between 2014-2017.
Trend in satisfaction with FLW visits	CHS	Annual change in log-odds of satisfaction with FLW visits between 2014-2017, based on the question, “Were you satisfied with the FLW visit”?
**Dependent**		
Birth preparedness	CHS	Increase and rate of change in the number of birth preparedness measures taken per block between 2014-2017.
Pregnancy registration	CHS	Rate of change in the block-level odds of pregnancy registration in the first trimester between 2014-2017.
Seeking care for complications	CHS	Rate of change in the block-level odds of seeking care for pregnancy complications between 2014-2017.
Antenatal care	CHS	Rate of change in the block-level odds of having at least four antenatal care visits during pregnancy between 2014-2017.
Immediate breastfeeding	CHS	Rate of change in the block-level odds of immediate breastfeeding between 2014-2017.
Dry cord care	CHS	Increase and rate of change in the block-level odds of dry cord care taken between 2014-2017.
Skin-to-skin care	CHS	Increase and rate of change in the block-level odds of skin-to-skin care between 2014-2017.
Age-appropriate complementary feeding	CHS	Increase and rate of change in block-level odds of age-appropriate complementary feeding between 2014-2017.
**Both dependent and independent**		
Occurrence of FLW visits	CHS	Increase and rate of change in the block-level odds of receiving a visit by a FLW to discuss mother’s and baby’s health.

SHRUG – Socioeconomic High-resolution Rural-Urban Geographic Platform for India; CFA – Comprehensive Facility Assessments; CHS – Community-based Household Surveys, FLW – frontline worker

We scaled the annual change in log odds of SHG membership, asset ownership, and satisfaction with FLW visits by 100 so that a unit change in the scaled variable was equivalent to a 0.01 change in the value of the actual variable.

### Analysis

#### Factors associated with increase in indicator levels

We applied this analysis to trends in the five indicators among the nine indicators analysed (**Table 2**) for which there was variation in the direction of change between 2014-2017: FLW visits, birth preparedness measures, skin-to-skin care, dry cord care, and age-appropriate frequency of complementary feeding [19]. We first tested the independent variables for their univariable association with the dependent variables. We used a *P*-value cut-off of 0.25 for inclusion in multivariable analysis and retained only the ones significant at the 0.05 level in the final model. We used multilevel logistic regression, accounting for clustering of block-level trends within districts. For all outcomes where FLW visits was an independent variable, we tested the interactions of each independent variable with FLW visits with a cut-off *P*-value of 0.05. We tested all models for nonlinear association with the baseline levels (2014) using a quadratic term. We did not include landscape as a covariate in the analysis of dry cord care or skin-to-skin care because we did not deem it as conceptually relevant.

#### Factors associated with the rate of change in indicators

For the second research question, we applied the method used by Baquero and Machado to all nine outcomes [27]. We re-ran hierarchical Bayesian models with spatiotemporal interaction terms, adding the covariates from the conceptual model. We first tested individual covariates separately and then tested their interactions with FLW visits in models for outcomes other than FLW visits. The best fitting model was the one with the lowest Akaike information criterion in comparison with the base model (ie, the model without covariates) and other covariate models. To estimate the explanatory power of the covariate on the spatiotemporal variation, we calculated the percentage change in variability of the spatiotemporal random effect as the standard deviation of spatiotemporal random effect of the base model without the covariate minus the standard deviation of the spatiotemporal random effect from the best fitting model with the covariate, divided by that of the base model and expressed as a percentage. A positive value meant that the covariate(s) was correlated with the spatial variation in indicator trend. A negative value indicated that the covariate was related to aspects of the outcome other than the spatial variation in its trend.

## RESULTS

### Description of block profiles

**Figure 2:**
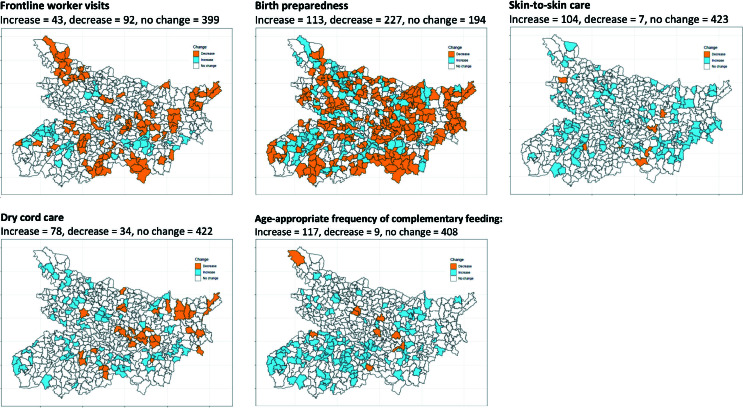
Change in indicator levels for five illustrative indicators with different directions of change in Bihar, India, 2014-2017.

**Table 3** shows the profile of Bihar’s 534 blocks with respect to contextual factors, including the coefficient of variability (CV) for each factor. Trend in satisfaction with FLWs varied the most across blocks (CV = 2.00). Birth preparedness, skin-to-skin care, and age-appropriate frequency of complementary feeding increased in approximately a fifth of the blocks, with FLW visits (8.1%) and dry cord care (14.6%) increasing in smaller numbers of blocks and birth preparedness decreasing in a relatively large proportion of blocks (42.5%) (**Figure 2**).

**Table 3:** Contextual variable profiles in 534 blocks in Bihar, India

Variable	Mean across 534 blocks	Standard deviation	CV	Minimum	Maximum
Average distance (km) to the nearest town with 500 000 population (remoteness)	138.39	105.10	0.76	8.07	587.31
Average luminosity (night lighting)	3.37	2.26	0.67	2.00	20.48
Average pixel values for forest coverage (forest coverage)	218.72	239.11	1.09	4.31	2841.23
Average daily hours of power available for domestic use (power availability for domestic use)	9.82	8.12	0.83	0.00	46.00
Percentage of villages/towns in a block that have any paved roads	0.52	0.27	0.52	0.00	1.00
Percentage of the population who are literate	0.54	0.09	0.17	0.31	0.84
Percentage of the population who belong to a scheduled caste or tribe	0.38	0.31	0.82	0.01	0.99
Trend in facility readiness score	7.45	14.91	2.00	-33.33	50.00
Annual change in average number of assets owned per block	0.09	0.02	0.22	0.01	0.17
Annual change in log-odds of the proportion of women who belong to a self-help group	0.41	0.02	0.05	0.34	0.48
Annual change in log-odds of the proportion of women who reported being satisfied with the frontline worker visit	0.19	0.01	0.05	0.14	0.22
Landscape index	0.00	1.00	NA	-3.13	5.41
Development index	0.00	1.00	NA	-2.04	3.63

CV – coefficient of variation

### Factors associated with increase in indicator levels

An increase in FLW visits between 2014 and 2017 was more likely in blocks with better night lighting (odds ratio (OR) = 1.23, 95% confidence interval (CI) = 1.01-1.51) and less likely in blocks with increasing wealth (OR = 0.80, 95% CI = 0.66-0.98) (**Table 4**). Increase in birth preparedness was significantly associated with increasing FLW visits (OR = 3.43, 95% CI = 1.15-10.21) while increase in dry cord care practice was significantly associated with increasing satisfaction with FLW visits (OR = 1.52, 95% CI = 1.10-2.11). Increase in age-appropriate frequency of complementary feeding was significantly associated with a higher development index (OR = 1.55, 95% CI = 1.16-2.06) and higher percentage of scheduled caste or tribe (OR = 3.21, 95% CI = 1.13-9.09). There was no significant interaction between FLW visits and any of the independent factors. None of the independent variables were significantly associated with skin-to-skin care. For all outcomes, increases between 2014 and 2017 were associated with lower levels in 2014, with evidence of a nonlinear association in the case of age-appropriate frequency of complementary feeding; the negative association with baseline values became stronger at higher indicator levels at baseline.

**Table 4:** Factors associated with increase in indicator levels (vs decrease or no change), Bihar, India, 2014-2017

Health indicator	Independent variable	Unadjusted odds ratio (95% CI)	Adjusted odds ratio (95% CI)
Frontline worker visits during pregnancy	Baseline level	0‧9 (0.87-0.94)	0.89 (0.86-0.93)
	Night lighting	1.15 (0.99-1.33)	1.23 (1.01-1.51)
	Trend in asset ownership	0.83 (0.71-0.98)	0.80 (0.66-0.98)
Birth preparedness	Baseline level	0.83 (0.80-0.86)	0.83 (0.80-0.86)
	FLW visit trend	2.07 (0.96-4.47)	3.43 (1.15-10.21)
Dry cord care	Baseline level	0.94 (0.92-0.95)	0.93 (0.92-0.95)
	Trend in satisfaction with FLW visit	1.34 (1.00-1.79)	1.52 (1.10-2.11)
Age-appropriate frequency of complementary feeding	Baseline level (10 percentage point increment)	1.02 (0.67-1.56)	1.00 (0.66-1.52)
	Baseline level quadratic term	0.95 (0.91-1.00)	0.95 (0.91-1.00)†
	Development	1.42 (1.09-1.87)	1.57 (1.17-2.09)
	% scheduled caste/tribe*	2.51 (0.95-6.67)	3.21 (1.13-9.09)

CI – confidence interval, FLW – frontline workers

*This variable was highly correlated with the remoteness variable and both were non-significant in the model when included together. Replacing it with remoteness did not affect the other covariates. Remoteness had a significant positive association (1.34, 95% CI = 1.02-1.77) per 100-mile increment.

†The “1.00” in the CI is due to rounding. The CI does not include 1 (*P* = 0.04).

### Factors associated with the rate of change in indicators

The rate of change in FLW visits explained 24% of the spatial variability in trends in immediate breastfeeding, while the rate of change in asset ownership explained 12% of the spatial variability in the rate of change in uptake of adequate antenatal care (**Table 5**). Rates of change of FLW visits and asset ownership taken together explained 15% of spatial variability in the rate of change in uptake of skin-to-skin care. Less variability (6%) in the rate of change in pregnancy registration and birth preparedness was explained by literacy and FLW advice on birth preparedness, respectively.

**Table 5:** Factors associated with the rate of change in indicator levels, 2014 - 2017, Bihar, India

Outcome	Covariate(s) that improved model fit the most	% change in spatiotemporal variation
FLW visit	Change in scheduled caste/tribe percentage	-15%
Pregnancy registration in the third trimester	Literacy	6%
Antenatal care: 4+ visits	Asset ownership	12%
Seeking care for pregnancy complications	Asset ownership	1%
Immediate breastfeeding	FLW visit	24%
Dry cord care	Asset ownership and FLW visit	-4%
Skin-to-skin care	Asset ownership and FLW visit	15%
Age-appropriate frequency of complementary feeding	Asset ownership interaction with FLW visit	-7%
Birth preparedness	Number of FLW birth preparedness measures advised	6%

FLW – frontline worker

## DISCUSSION

We set out to investigate factors associated with the geographical variation in direction and magnitude of trends over time in key RMNCHN practices targeted by the *Ananya* programme to understand contextual determinants of programme impact. We found that improvements in indicators are not linked to health system-specific programmatic interventions alone, highlighting particularly influential contextual factors that can be linked to improvements in health outcomes. An increase in FLW visits was associated with more night lighting, while improvements in age-appropriate frequency of complementary feeding were associated with the level of development as defined by power availability, night lighting, and literacy, collectively. Improvements in dry cord care practices and birth preparedness were more closely linked with factors related to FLW visits that were directly targeted by the programme. In relation to the rate of improvements, asset ownership and FLW visits appeared to be the most influential.

Our findings shed new light on the influence of contextual factors that are typically not the focus of health programmes. We offer uniquely quantitative evidence linking night lighting with increase in FLW visits. It is possible that night lighting is a proxy for the level of development or socioeconomic status, however, this appears to be unlikely since the other measures related to the latter were not significantly associated with the outcome. It is possible that better night lighting offers FLWs longer hours over which to conduct visits; anecdotally, solar lanterns distributed to ASHAs through a non-governmental organisation programme enabled them to conduct night visits in homes that did not have electricity [28]. It is also possible that well-lit areas convey a perception of safety. Although there is no concrete evidence from Bihar or India in general, some rigorous controlled studies in the UK showed that improved street lighting reduced fear of crime, although the evidence remains mixed [29]. The negative association of increases in FLW visits with wealth appears to affirm previous findings that FLW visits tended to favour the most disadvantaged, as anticipated for a programme which aimed to increase equity in programme coverage and outcomes [20]. Improvements in age-appropriate frequency in complementary feeding are understandably reliant on many other factors besides women’s knowledge of how often children should be fed and are closely linked with food security, competing priorities [30], and social norms [31]. The close link between improvements in this indicator and the level of development in the block is therefore not surprising. Wealth is another contextual factor outside the health system that was influential in terms of rates of change in antenatal care and skin-to-skin care, indicating that even when the level of an indicator is increasing consistently across geographies, poverty can be a barrier to accelerated change.

Trust in FLWs was previously shown to be critical for acceptance of maternal and child health services [32,33]. We measured satisfaction, a correlate of trust [34], and found that, wherever the satisfaction with the FLW visit was increasing, the practice of dry cord care increased as well. Application of various materials to the cord stump is a common practice that may be resistant to change due to social factors [35]. A connection with the FLWs’ level of satisfaction may reflect more widespread community-level trust in their FLWs, emphasising its importance, particularly when information provided by FLWs is the only resource needed for behavioural change as is the case with dry cord care.

Another important issue that directly relates to the capacity to benefit from programme interventions is the association between the indicator level at the beginning of scale-up and likelihood of improvements. We initially expected a potentially nonlinear association. We assumed that indicators may have lower levels at the beginning due to pervasive structural barriers against improvement, impeding progress in areas with lower baseline levels during Ananya scale-up [10]. On the other hand, indicators with relatively high levels at the beginning of scale-up may have already fulfilled their capacity to benefit and therefore may not respond to further programme inputs. We consistently found, however, that improvements were more likely at lower levels with no evidence of a nonlinear association for the majority of indicators. It therefore appears that there was minimal resistance to improvement once relevant contextual non-health drivers were also addressed.

This work is the latest in a series of analyses aimed to describe and explain the Ananya programme’s impacts in Bihar [10-20,36-39]. Our intention was not to re-examine the already well-established links between health and social determinants, but to examine how these links are influencing the outcome of the programme scale-up over time and in different areas, and the need for approaches to be more conscious of the existing ecosystem in which health programmes are embedded. The results carry key implications for health programming and intervention scale-up. Scaling up of the interventions via the BTSP had a key role in ensuring widespread benefits in the state, and health programmes should continue to improve health-specific community outreach with visits and health advice and target marginalised groups with interventions. However, they should also consider modifying intervention design and mix to fit the local context, interrogating and confirming relevant pathways via further research. While addressing health directly, programmes may expand the rate, magnitude, predictability, and sustainability of impact through collaborations and advocacy efforts that extend beyond the health sector to influence modifiable contextual factors. Although the data we used came from one state in India, similar contextual factor profiles and programming efforts are present in other states as well as other low- and middle- income countries, and therefore our results can be reasonably generalised to similar settings.

Our findings also carry implications for monitoring and evaluation. They underscore the importance of collecting data at the lowest geographical and administrative level possible to carry out comparative assessments of programme impact, an issue that is increasingly being highlighted [40]. With this comparative view, geospatial analysis can be an additional tool for defining worst and best performance based on agreed upon definitions, refining local targets, and modifying intervention mix and approach accordingly, and identifying reasons for trends, including stagnation in some areas. Finally, accounting for structural/contextual determinants of programme impact is not a new concept, but may often be hard to implement in practice due to lack of data. Our findings illustrate that the collection of geospatial data on environmental and other contextual factors is a necessary investment that will also help fuel achievement of the broad range of SDGs.

Our analysis has several strengths. As products of Hierarchical Bayesian models, our trend estimates account for instability in block-level estimates due to small sample sizes at that level. Another strength is the ability to use data from external sources, linking contextual and other environmental factors with health trends at the block level. In the absence of panel data with individual participant follow-up during the programme, our study’s approach is the closest possible to constructing and explaining a longitudinal profile of indicator change.

Our study also has important limitations. We faced limited availability of geospatial data at the exact time points required, including data on programme inputs and resources. We also remain limited by the assumptions we made in our Bayesian modelling and the self-reporting nature of the survey data underlying outcome measures. Finally, we cannot confirm causal links in the context of Bihar solely based on these results.

## CONCLUSIONS

We consider this work to be an early step in a series of future efforts to collate additional data, update trends in indicators, and further understand the social and contextual determinants of change in light of the programme’s health interventions. The results show that much can be gained from investments in this analytical approach and in improving data collection, particularly for contextual factors. Further work can explore the relative contribution of individual against contextual effects, target locally agreed upon priority indicators such as neonatal and child mortality, and apply more specific definitions for improvements in indicator levels such as global or local targets.

## Additional material:


Online Supplementary Document
