# Supplementary material for: Improving the precision of maternal, newborn, and child health impact through geospatial analysis of the association of contextual and programmatic factors with health trends in Bihar, India
Source: J Glob Health. 2022 Nov 23;12:04064. doi: 10.7189/jogh.12.04064 (PMC9679706; doi:10.7189/jogh.12.04064)

Supplement

Improving precision of maternal and child health impact through geospatial analysis of the association of contextual and programmatic factors with health trends in Bihar, India

Safa Abdalla,[1] Emma Pair,[1] Kala Mehta,[2] Victoria Ward,[1] Tanmay Mahapatra,[3] Gary L Darmstadt[1]

[1]Department of Pediatrics, Stanford University School of Medicine, Stanford, CA, USA
[2]Department of Epidemiology and Biostatistics, University of California San Francisco, San Francisco, CA, USA
[3]CARE India, Patna, India

Correspondence: Gary L. Darmstadt, MD, Department of Pediatrics, Stanford University School of Medicine, 1701 Page Mill Road, Stanford, CA 94305, USA, gdarmsta@stanford.edu

Supplementary Table S1. Description of maternal and child health indicators from Community-based Household Surveys, Bihar, India, 2014-2017

Indicator	Child age group (months)	Indicator description
Any antenatal frontline worker (FLW)* visit to discuss mother's and baby's health	0-2	FLW ever visited the mother to talk to her about her health or her baby's health
Number of birth preparedness measures taken	0-2	Based on the following measures: saving money for delivery or emergency, chosen facility for deliver, chosen facility in emergency situation, arranged transportation to facility, arranged for someone to accompany them, identified someone who will take care of the baby immediately after delivery, identified someone who will donate blood in case of emergency, identified and informed anyone who would assist with delivery, and prepared Disposable Delivery Kit, a new blade, clean thread, soap, a clean cloth for the child, and clean pads for her.
Pregnancy registration in the third trimester	0-2	Mother registered her pregnancy no later than the first three months of pregnancy.
Sought care for complications	0-2	Mother consulted anybody or sought treatment if she had experienced excessive bleeding or convulsions
4+ antenatal care visits	0-2	Had four or more antenatal care visits during last pregnancy
Skin-to-skin care	0-2	Mother was advised by anyone at any time during or after delivery to keep the baby naked on her bare chest, next to your skin, and practiced keeping the baby in that position immediately after delivery/during the stay in the hospital (for facility deliveries)
FLW advised exclusive breastfeeding	0-2	FLW told the mother that the baby should not be given anything other than breastmilk up to the age of six months
Immediate breastfeeding	0-2	Breastfeeding initiated within one hour after birth
Age appropriate frequency of complementary feeding	9-11	Baby given cereal-based semisolid food 3-4 times per day

*Frontline workers include Accredited Social Health Activists (ASHAs), Anganwadi Workers (AWWs) and Auxiliary Nurse Midwives (ANM)

Supplementary Table S2. Factor loadings for each of the variables included in the Principal Components Analysis

Variable	Factor 1	Factor 2
Distance to nearest town with 500k population	0·37	-0·68
Average night lighting	0·35	-0·01
Forest coverage	0·0	0·61
% literate	0·78	-0·06
% power availability for domestic use	0·83	-0·12
% land with paved roads	0·03	0·82

Supplementary Figure S1. Correlations between the dependent and independent variables, antenatal care – ANC, frontline worker – FLW.

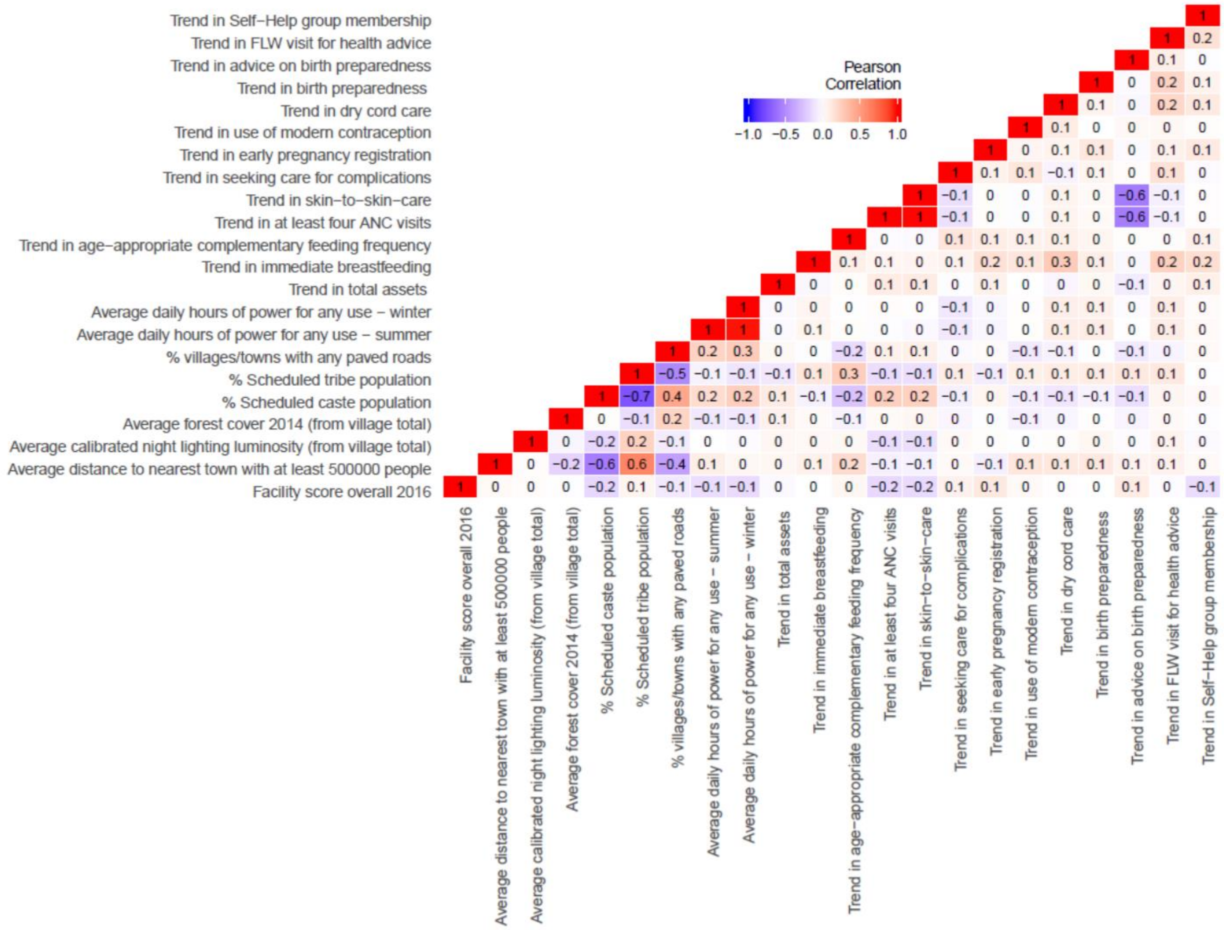

Supplement: Online Supplementary Document [file jogh-12-04064-s001.pdf]
